# Effects of Estradiol Therapy on Resting-State Functional Connectivity of Transgender Women After Gender-Affirming Related Gonadectomy

**DOI:** 10.3389/fnins.2019.00817

**Published:** 2019-08-07

**Authors:** Maiko A. Schneider, Poli M. Spritzer, Luciano Minuzzi, Benicio N. Frey, Sabrina K. Syan, Tayane M. Fighera, Karine Schwarz, Ângelo B. Costa, Dhiordan C. da Silva, Cláudia C. G. Garcia, Anna M. V. Fontanari, André G. Real, Maurício Anes, Juliana U. Castan, Fernanda R. Cunegatto, Maria I. R. Lobato

**Affiliations:** ^1^Gender Identity Program (PROTIG), Psychiatric Service, Hospital de Clínicas de Porto Alegre, Porto Alegre, Brazil; ^2^Mood Disorders Program, Women’s Health Concerns Clinic, St. Joseph’s Healthcare Hamilton, Hamilton, ON, Canada; ^3^Department of Psychiatry and Behavioural Neurosciences, McMaster University, Hamilton, ON, Canada; ^4^Department of Physiology, Universidade Federal do Rio Grande do Sul, Porto Alegre, Brazil; ^5^Division of Endocrinoloy, Hospital de Clínicas de Porto Alegre, Porto Alegre, Brazil; ^6^Neuroscience Graduate Program, McMaster University, Hamilton, ON, Canada; ^7^Peter Boris Centre for Addictions Research, McMaster University, Hamilton, ON, Canada; ^8^Graduate Program in Psychology, Pontifícia Universidade Católica do Rio Grande do Sul, Porto Alegre, Brazil; ^9^Post-Graduation Program, Universidade Federal do Rio Grand do Sul, Porto Alegre, Brazil; ^10^Medical Physics and Radiation Protection Service, Hospital de Clínicas de Porto Alegre, Porto Alegre, Brazil; ^11^Psychology Service, Hospital de Clínicas de Porto Alegre, Porto Alegre, Brazil; ^12^Psychiatric and Forensic Medical Service, Hospital de Clínicas de Porto Alegre, Porto Alegre, Brazil

**Keywords:** gender dysphoria, hypogonadism, estradiol, functional connectivity, sensorimotor cortex, putamen, thalamus

## Abstract

An extreme incongruence between sex and gender identity leads individuals with gender dysphoria (GD) to seek cross-sex hormone therapy (CSHT), and gender-affirming surgery (GAS). Although few studies have investigated the effects of CSHT on the brain prior to GAS, no studies in the extant literature have evaluated its impact during hypogonadism in post-GAS individuals. Here, we aimed to evaluate the effects of estradiol on resting-state functional connectivity (rs-FC) of the sensorimotor cortex (SMC) and basal ganglia following surgical hypogonadism. Eighteen post-GAS (male-to-female) participants underwent functional magnetic resonance imaging (fMRI) and neuropsychiatric and hormonal assessment at two time points (t1, hormonal washout; t2, CSHT reintroduction). Based on the literature, the thalamus was selected as a seed, while the SMC and the dorsolateral striatum were targets for seed-based functional connectivity (sbFC). A second sbFC investigation consisted of a whole-brain voxel exploratory analysis again using the thalamus as a seed. A final complementary data-driven approach using multivoxel pattern analysis (MVPA) was conducted to identify a potential seed for further sbFC analyses. An increase in the rs-FC between the left thalamus and the left SCM/putamen followed CSHT. MVPA identified a cluster within the subcallosal cortex (SubCalC) representing the highest variation in peak activation between time points. Setting the SubCalC as a seed, whole-brain analysis showed a decoupling between the SubCalC and the medial frontal cortex during CSHT. These results indicate that CSHT with estradiol post-GAS, modulates rs-FC in regions engaged in cognitive, emotional, and sensorimotor processes.

## Introduction

Gender dysphoria (GD) is characterized by incongruence between the sex assigned at birth and the gender identity. People who experience profound gender incongruence are referred for cross-sex hormone therapy (CSHT) and gender-affirming surgery (GAS) (Hembree et al., 2017). Apart from their effects on the body, sex hormones are also known to be neuroactive (Zheng, 2009), and several studies have demonstrated the impact of CSHT on brain structure and functional connectivity (Rametti et al., 2012; Mueller et al., 2016, 2017). Thus far, most of the studies evaluating the effects of CSHT were conducted before GAS (Nguyen et al., 2018); as of yet, little is known about its effects on the brain following gonadectomy. Although there are no estimates about the worldwide numbers of gonadectomies performed over previous decades, a 12-year cohort called STRONG (The study of transition, outcomes, and gender) estimated a total of 363 orchiectomies/oophorectomies in Georgia, Northern California and Southern California (United States) (Quinn et al., 2017). Another survey in Korea reported a total of 115 GAS; however, that report specified neither how many included gonadal removal nor the time window in which they had been performed (Lee et al., 2018). In our outpatient program for GD, from 2000 to 2016, we completed 174 GAS including orchiectomy (Schwarz et al., 2017). Considering these numbers, it is a matter of public health concern to better investigate the impact of CSHT on a transgender person’s brain after completion of gonadectomy.

Following removal of the gonads, transgender individuals experience hypogonadism-related symptoms similar to those presented by menopause-transitioning women, such as hot flushes, if CSHT is not continued. A plausible hypothesis for the development of these symptoms is the disruption of the functional connectivity between the thalamus (secondary neuron) and the primary sensorimotor cortex (SMC) (tertiary neuron) as a result of the decline of circulating sex hormones. Anatomically, the thalamus is a region contiguous to the SMC and the basal-ganglia, and functionally, it functions as a modulator of sensory and motor synapses (Behrens et al., 2003). The thalamus also conveys and integrates sensory input within the thalamocorticostriatal circuitry (Butler et al., 1992; Herrero et al., 2002), and its involvement in the stimulus-controlled processing stream within this circuitry has also been proposed in a theoretical frame (Alloway et al., 2017). Of note, the thalamus, basal ganglia and the SMC express a great number of estrogen receptors in the human brain (Osterlund et al., 2000; Osterlund and Hurd, 2001), which contributes to the hypothesis that these structures are prone to being affected by a deficiency in sex hormones.

With regard to functional connectivity, Kenna et al. (2008) had already demonstrated that menopause-related estrogen decline was associated with a disruption in the integrity of the thalamostriatal synapses (Kenna et al., 2008). In that experiment, using [18F] fluorodeoxyglucose positron emission tomography (18FDG-PET), the investigators found an increase in the functional connectivity/coupling of this circuit after estrogen administration. This finding suggested that circulating estradiol was a modulator of the sensorimotor circuitry, contributing to the integrity, and function of thalamic synapses after ovarian failure. Moreover, estradiol replacement therapy was associated with an improvement in fine motor ability during menopause in a clinical study from Bayer and Hausmann (2010) (Bayer and Hausmann, 2010). These results are in line with experimental data from animal models that showed neuroprotective effects of estrogen therapy in the face of more adverse conditions such as stroke and ischemia (Ardelt et al., 2012; Carpenter et al., 2016).

Resting-state functional connectivity (rs-FC) is a neuroimaging technique used to map the brain that is related to blood oxygen level-dependent (BOLD) imaging (Hart et al., 2016; Qiu et al., 2017) that has been applied to investigate several neurological and psychiatric conditions (Uddin et al., 2009; Chen and Etkin, 2013). When two or more brain regions present with temporally correlated changes in the BOLD signal it implies a “functional connectivity.” Discrete brain regions may have correlated/coupled connectivity or anticorrelated/decoupled connectivity. Investigating how the brain works in the resting state is useful to better understand neuroplastic mechanisms in response to different physiological and pathological conditions. Therefore, rs-FC can be helpful to better understand how CSHT affects functional connectivity after gonadectomy is performed in transgender people.

As it is still a largely unexplored field, we designed the present study to investigate the effects of CSHT on transgender women’s brain functional connectivity after gonadectomy-related GAS. The principal aim was to determine the effects of estradiol on the primary SMC and the striatum of transgender women. To fulfill this objective, region-of-interest to region-of-interest (ROI-to-ROI) and seed-to-voxel rs-FC analyses were performed. First, the thalamus was set as a seed region, while the striatum and the SMC were used as targets in the ROI-to-ROI approach. The rationale for using the thalamus as a seed was its already demonstrated anatomical and functional connection to these structures (Giménez-Amaya et al., 1995; Herrero et al., 2002; Behrens et al., 2003; Hwang et al., 2017). We hypothesized that hypogonadism correction following estradiol administration increases the rs-FC between the thalamus, primary SMC, and the dorsolateral striatum. Next, an additional exploratory seed-to-voxel analysis was done setting the thalamus as a seed, while whole-brain voxels were defined as targets. Last, as a secondary objective, a complementary functional data analysis using whole-brain multivoxel pattern analysis (MVPA) generated a seed for a last seed-to-voxel exploratory analysis.

## Materials and Methods

### Patients

Eighteen transgender women (male-to-female) who had already completed GAS were selected from a specialized outpatient program for gender incongruence (Programa Ambulatorial Transdisciplinar de Identidade de Gênero – PROTIG, Hospital de Clínicas de Porto Alegre, Porto Alegre, Brazil). The recruitment was done from February 2017 to February 2018. Inclusion criteria were (1) GD diagnosis according to the DSM-5, (2) age between 18 and 59 years, and (3) at least 1 year post-GAS, without surgical repairs in the meantime. Exclusion criteria were (1) use of antidepressant, mood stabilizer, antipsychotic, or anticonvulsant drugs in the last 90 days; (2) presence of depressed mood, anxiety, panic or obsessive-compulsive disorder, as assessed through clinical interviews conducted by a psychiatrist and a psychologist in accordance with the DSM-5; (3) presence of current substance use disorder; (4) history of head injury with loss of consciousness or neuroimaging sequelae; or (5) presence of any contraindication to MRI. One participant was excluded due to brain anatomical variation. Anxiety and mood disorders were chosen as exclusion criteria since these mental health conditions are known to affect functional brain connectivity (Yang et al., 2017; Bai et al., 2018; Kunas et al., 2018), and they could potentially represent confounds to this investigation. All the patients were right-handed according to the Edinburg Handedness Inventory (Veale, 2014). After completing the initial screening, participants ceased CSHT for at least 30 days in order to promote a standardized hormonal washout among all participants. On the last day of the washout period (t1), all participants underwent MRI scans, as well as laboratory, and mood/anxiety assessments. Notably, this relatively short window took into consideration ethical concerns regarding depriving patients of hormonotherapy for longer periods. Afterward, patients were put back on CSHT regimes according to the clinical protocols for more 60 (±1 week variance for the visit), using exclusively estradiol formulations (Hembree et al., 2017). Then, the participants completed the same MRI, laboratory and mood/anxiety assessments on the 60th day of CSHT (t2) as they did at t1.

Sample size was estimated based on the study of Kenna et al. (2008). We initially expected to recruit 20 volunteers, although interim analysis revealed sufficient power analysis with 17 individuals. This project was approved by the Ethics Review Board of the Hospital de Clínicas de Porto Alegre (CEP 15-0199). All participants gave written informed consent according to the Declaration of Helsinki.

### Neuropsychological Evaluation

At the first and second time points (t1 and t2), participants were evaluated using the Hamilton Rating Scales for anxiety (HAM-A), and depression (HAM-D) (Hamilton, 1958, 1959). Paired *t*-tests were used to compare differences between mean HAM-A/HAM-D scores between time points. Although participants did not present with mood and anxiety disorders when admitted to the study, these scales were intended to detect potential somatic anxiety symptoms frequently reported by subjects during hypogonadal conditions or CSHT adjustment, at a statistical threshold of *p* < 0.05.

### MRI Acquisition Protocol

All subjects were scanned in a Philips Ingenia 3.0 T MR system (Best, Netherlands, 2015) using a 32-channel head coil. An rs-fMRI and a high-resolution structural sequence were acquired from each individual before and after CSHT. The rs-fMRI sequence was an echo planar imaging (EPI) with 36 slices of T2^*^ weighted images with the following acquisition parameters: TE = 30 ms, TR = 2000 ms, flip angle 90°, 80 × 80 matrix, in-plane voxel size = 3.5 mm × 3.5 mm, slice thickness = 3.5 mm, gap slice spacing = 0.35 mm, FOV = 240 mm, lasting for 6:12 min. Structural images were acquired using a T1-weighted 3D magnetization prepared rapid acquisition with gradient echo (MPRAGE) sequence with 200 sagittal slices, flip angle = 8°, TE = 3.9 ms, TR = 8.5 ms, TI = 900 ms, flip angle = 8°, 256 × 256 matrix, matrix size = 272 × 272, in-plane voxel size 0.94 mm × 0.94 mm, slice thickness 0.94 mm, no gap, and FOV = 256 mm.

### Laboratory Analysis

Venous blood samples were collected between 8 a.m. and 10 a.m. on the same day MRI scans were done. Estradiol was measured by electrochemiluminescence immunoassay (ECLIA, Roche Diagnostics, Mannheim, Germany), with assay sensitivity of 5.0 pg/mL and intra- and interassay CV of 5.7 and 6.4%, respectively. Follicle-stimulating hormone (FSH) and luteinizing hormone (LH) were measured by chemiluminescence immunoassay (Centaur XP, Roche Diagnostics, Mannheim, Germany), with sensitivity of 0.10 mIU/mL, intraassay coefficient of variation (CV) of (<3% and interassay CV of <5%).

### Functional MRI Data Preprocessing

Resting-state functional connectivity data were analyzed with the CONN toolbox (version 18) (Whitfield-Gabrieli and Nieto-Castanon, 2012). The preprocessing included the following steps: functional realignment and unwarp for subject motion estimation and correction, functional images centering on (0, 0, 0) MNI coordinates and slice-time correction. ART-toolbox was used for functional outlier detection, using an intermediate setting of 97th percentile and a 0.9 mm threshold for motion. The structural and functional images were segmented and normalized with a simultaneous segmentation and normalization for gray/white matter and CSF. Structural and functional target resolutions were 1 and 2 mm, respectively. Functional smoothing was used to spatial convolution with Gaussian kernel of 8 mm. First-level covariates derived from this process were checked for quality of the data; therefore, motion and outliers could be added as first-level covariates. A maximum of 35 invalid scans due to excessive motion per condition was permitted, and no participant had to be excluded for this reason. Principal component analysis (PCA) with additional anatomical component-based noise correction method (*aCompCor*) (Behzadi et al., 2007; Whitfield-Gabrieli and Nieto-Castanon, 2012; Muschelli et al., 2014) was applied to obtain signal from the CSF and WM. The Harvard-Oxford Atlas (Desikan et al., 2006) was used to extract signal from the regions of interest using average time series extraction. The confounds during the denoising process included signals from white matter and CSF, slice realignment and scrubbing. A bandpass filter (0.008–0.09) using linear detrending was applied during noise filtering. Functional connectivity analysis was carried out using a weighted general linear model, with a zero-lagged bivariate linear correlation in order to prepare data for seed-based connectivity analysis. A block design (estradiol > washout) was defined to compare conditions on second-level analyses after the completion of the preprocessing steps.

Additionally, a voxel-to-voxel first-level analysis was prepared to conduct further data-driven analysis using group-MVPA. MVPA is a method that uses a low-dimensional representation of the connectivity values between whole brain voxels in order to identify reproductible patterns (voxel-to-voxel features) that differentiate two or more experimental conditions (Mahmoudi et al., 2012). For each voxel tested as seed a representational matrix for whole brain connectivity is build, until all voxels have been tested as seeds. Functional data were prepared using the first 4 principal components of the PCA and 64-voxel dimensionality reduction. An omnibus test was carried out to compared connectivity patterns between the two experimental conditions. This was a complementary second-level analysis in order to obtain a new seed from the data for *post hoc* rs-FC exploratory analysis (Norman et al., 2006; Peelen and Downing, 2007). As we intended to characterize connectivity patterns between the data-driven seed and the rest of the brain (*post hoc* analysis), no additional cross-validation is needed for the MVPA algorithm (Whitfield-Gabrieli and Nieto-Castanon, 2012; Beaty et al., 2015; Thompson et al., 2016).

### Statistics

Clinical and laboratory data were analyzed with R software^1^ (version 3.4.1). Normal distribution for clinical and laboratory data was assessed by the Shapiro-Wilk test.

### Seed-Based FC Using *a priori* Defined Seed (ROI-to-ROI and Seed-to-Voxels)

First, ROI-to-ROI analysis was conducted defining the thalamus as a seed (Kenna et al., 2008), while the SMC and the dorsolateral striatum were defined as targets (Lanciego et al., 2012; Borich et al., 2015; Carpenter et al., 2016; Nota et al., 2017). A contrast between t1 and t2 (estradiol > washout) was designed to estimate connectivity differences (rs-FC) between the conditions; furthermore, we proceeded with a multiple regression analysis to test whether there is a relationship between changes in rs-FC and changes in estradiol serum levels between time points. Age and variation in anxiety scores between time points were controlled in both analyses. Including changes in anxiety scores between time points was considered a reasonable approach, since they could potentially interfere with the functional connectivity of the sensory cortex. Compared to the HAM-D scale, the HAM-A includes more somatic domains, which are helpful to identify the impact of hypogonadism symptoms on the sensorimotor connectivity. Multiple comparisons were corrected for false discovery rate (p-FDR <0.05) according to the number of functional connectivities tested for the ROI-to-ROI approach.

Second, a seed-to-voxel whole-brain analysis was conducted setting the thalamus as a seed, while all brain voxels were set as targets to estimate connectivity differences between the two time points (estradiol > washout). As in the ROI-to-ROI approach, age and changes in anxiety scores between time points were controlled. We also tested for the relationship between changes in estradiol levels and changes in rs-FC between time points, following similar approach as on ROI-to-ROI analysis. Multiple comparisons were corrected for using a peak height threshold at *p* < 0.001 plus a sequential cluster-size p-FDR thresholded at *p* < 0.05, both two-sided.

*Post hoc* analyses were done to test for the effects of prior lifetime exposure to CSHT on the rs-FC, since it could influence the rs-FC responsiveness after estradiol reintroduction, as well as for lateralization effect on functional connectivity when comparing left, and right thalami on whole-brain seed-based analysis.

For both seed-based analyses, we conducted supplementary exploratory analysis to investigate whether intraindividual variations in anxiety and depression (changes in HAM-A/HAM-D scores between time points) are related to changes in rs-FC.

### Seed-Based FC Analysis Using a Data-Driven Seed

Finally, we ran a complementary whole-brain seed-to-voxel analysis using the seed derived from a data-driven approach (group-MPVA) in order to estimate connectivity differences between the two conditions. Statistical significance for this whole-brain seed-to-voxel analysis was thresholded at <0.001 for peak height, with further cluster-size correction at 0.05 for FDR (cluster-size p-FDR <0.05, two-sided).

Briefly, group-MVPA implemented by CONN uses a machine learning algorithm to classify the voxels that exhibit the greatest variability in peak activation between two or more conditions. Here, a contrast between estradiol (CSHT) and the washout condition was designed to investigate changes in functional connectivity following reintroduction of estradiol regimes (estradiol > washout). Statistical significance for group-MVPA was thresholded at <0.001 for peak height, with additional cluster-size correction at 0.05 for FDR (cluster-size p-FDR <0.05), both two-sided. Age differences between subjects were controlled. When conducting posterior seed-based connectivity analysis using the cluster obtained from the MVPA as a seed, HAM-A variation between time points was added as a covariate, and following the same approach used for the other seed-based analysis. Again, we investigated whether intraindividual changes in HAM-A/HAM-D are related to changes in whole-brain rs-FC.

## Results

### Sociodemographic Features

Table 1 presents clinical and hormonal characteristics of the sample. The mean age of participants was 40.2 years (±8.9 years); although the inclusion criteria were from 18 to 59 years old, participants were aged between 27 and 59. The mean for years of education was 10.2 (±3.3 years), and the mean for lifetime exposure to CSHT was 17.71 years (±8.62 years). Median time elapsed from GAS to t1 was 2 years, interquartile interval (IqI) between 1 and 4 years. The mean of number of days off CSHT was 35.86 (±3.32 days), and the number of days on CSHT between time points was 66.1 (±5.49). All participants were right-handed. As expected, estradiol, LH and FSH serum levels changed significantly during CSHT.

**TABLE 1:** Clinical and laboratory characteristics of the sample.

**Clinical and laboratory characteristics of the sample**			
Age (years, mean/sd)	41.59 (8.66)		
Age beginning CSHT (years, mean/sd)	22 (7.98)		
Lifetime exposure to CSHT (years, mean/sd)	17.71 (8.62)		
Time post-GAS (years, median/IqI)	2 (1–4)		
Time off CSHT (days, mean/sd)	35.86 (3.32)		
Time on CSHT after t1 (days, mean/sd)	66.1 (5.49)		
Handedness (mean/sd)	98.94 (4.36)		

	**t1**	**t2**	***p-value***

E2 pg/mL (median/IqI)	<5.0 (0–0)	93.23 (25.8–135.30)	0.0002^*^
Testosterone ng/mL (mean/sd)	0.07 (0.10)	0.11 (0.15)	0.43
LH mIU/mL (mean/sd)	50.85 (22.01)	30.7 (22.99)	0.10
FSH mIU/mL (mean/sd)	91.75 (38.01)	56.56 (33.61)	0.02^*^
HAM-D (median/IqI)	4.0 (1–10)	2 (1–6)	0.51
HAM-A (median/IqI)	5 (1–12)	2 (1–10)	0.16

Pre and post comparisons were done with paired *t*-test or Wilcox test. Interpretation of handedness scale: scores <−40 indicates predominantly left handed; scores between −40 and +40 indicates ambidextrous; scores >+40 indicate predominantly right handed. GD, gender dysphoria; CD, cross-dressing; CSHT, cross-sex hormone therapy; SHBG, sex hormone binding globulin; LH, luteinizing hormone; FSH, follicle-stimulating hormone; DHEA-S, dehydroepiandrosterone sulfate; FSIQ, full-scale intelligence quotient; HAM-D, hamilton depression rating scale; HAM-A, hamilton anxiety rating scale; IqI, interquartile interval; sd, standard deviation; t1: washout, t2, CSHT conditions; ^*^, significant at *p* < 0.05.

### Clinical Assessment

Anxiety and depression scores after the washout period (t1) and after the reintroduction of estradiol (t2) are presented in Table 1. No significant changes in HAM-D or HAM-A scores were observed after CSHT reinstitution. Supplementary Figure S1 illustrates individual changes in anxiety and depression scores between time points (t2 – t1).

### Resting-State Functional Connectivity

#### Seed-Based FC Using *a priori* Defined Seeds

**FIGURE 1:**
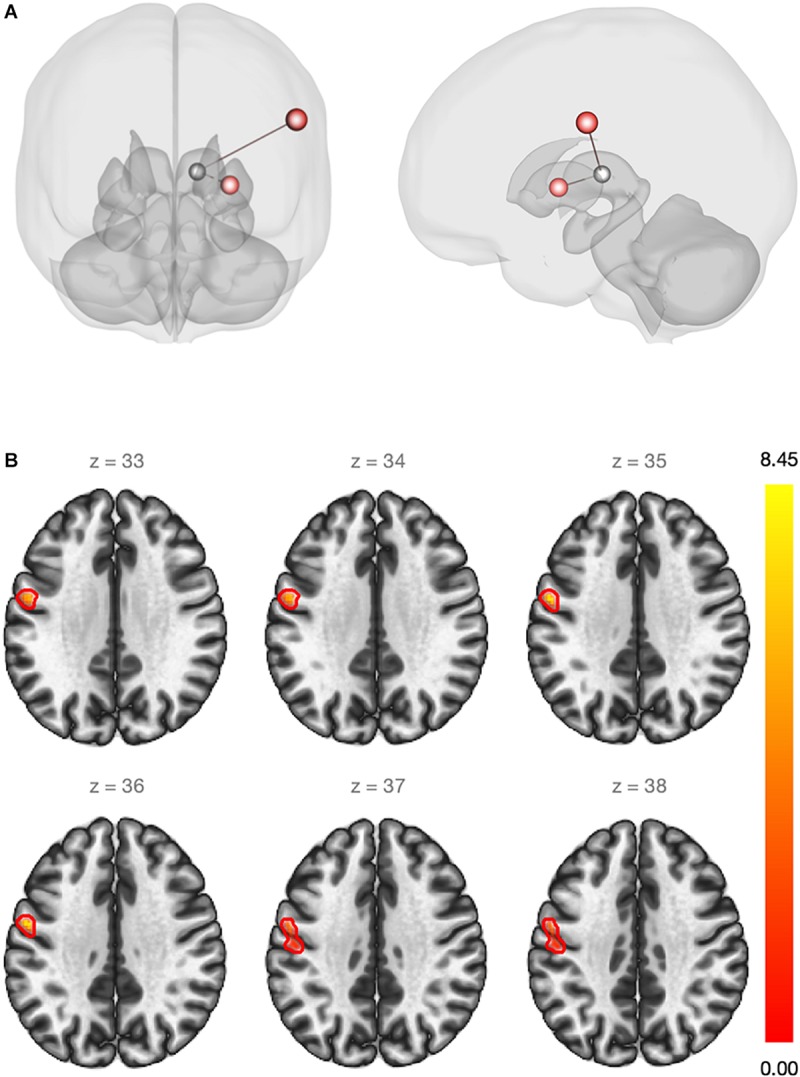
**(A)** Red regions show an increase in the rs-FC between the left thalamus and the left sensorimotor cortex (beta = 0.20)/left putamen (beta = 0.14) after estradiol therapy (ROI-to-ROI analysis). Statistical significance thresholded at p-FDR <0.05. **(B)** Whole-brain seed-to-voxel functional connectivity analysis. Red cluster indicates increased rs-FC between the left thalamus (seed) and voxels of the pre and post-central gyri (beta = 0.21). Cluster size p-FDR <0.0042. Color bar shows statistical significance.

First, the ROI-to-ROI analysis revealed a significant group effect of estradiol (CSHT) on rs-FC (Table 2). Compared to the washout, 60 days of estradiol therapy was associated with a significant increase in connectivity (coupling) between the left thalamus and the left SMC (*p* = 0.016), as well as a coupling between the left thalamus and the left putamen (*p* = 0.026) (Figure 1A). There was no significant relationship between estradiol levels and changes in the rs-FC regarding the seed and targets when running the multiple regression analysis. As well, there was no significant correlation between intraindividual changes in HAM-A/HAM-D and rs-FC between thalamus and the respective targets.

**TABLE 2:** Region-of-Interest to Region-of-Interest analysis.

**Seed**	**Target**	**Beta**	**T (14)**	**p-unc**	**p-FDR**
L thalamus					
	L lateral sensorimotor	0.20	3.86	0.002	0.016^*^
	R lateral sensorimotor	0.13	2.62	0.020	0.060
	S sensorimotor	0.06	0.93	0.286	0.474
	L putamen	0.14	3.26	0.006	0.026^*^
	R putamen	0.06	0.94	0.264	0.474
	L caudate	0.13	1.49	0.158	0.356
	R caudate	0.10	1.17	0.262	0.472
	L pallidum	0.05	0.76	0.458	0.516
	R pallidum	0.03	0.66	0.519	0.519
R thalamus	L lateral sensorimotor	0.20	2.87	0.012	0.086
	R lateral sensorimotor	0.17	2.65	0.191	0.086
	S sensorimotor	0.04	0.73	0.475	0.590
	L putamen	0.10	1.61	0.130	0.292
	R putamen	0.06	1.30	0.187	0.337
	L caudate	0.06	0.73	0.475	0.590
	R caudate	0.09	1.10	0.290	0.435
	L pallidum	0.11	2.31	0.036	0.109
	R pallidum	–0.03	–0.45	0.658	0.658

Estimated connectivity differences in the resting-state functional connectivity comparing estradiol and washout time points (t2 > t1). Analyses were controlled for age differences and changes in anxiety scores according to the Hamilton Inventory. Beta indicates the average difference on the connectivity values across the regions of interest. L, left; R, right; S, superior; Rs-FC, resting-state functional connectivity; unc, uncorrected for multiple comparisons; FDR, false discovery rate; ^*^, statistical significance after FDR <0.05.

Second, the whole-brain seed-to-voxel analysis using the thalamus as a seed demonstrated an increase in the rs-FC between the left thalamus and a cluster of voxels from the left primary motor and sensory cortices (coordinates: −56 −04 36; cluster size: 239 voxels; peak height uncorrected: 0.00001; size p-FDR: 0.004) (Figure 1B). Again, there was no statistically significant relationship between changes in estradiol levels and changes on rs-FC. Supplementary Material contains spatial connectivity maps for both time points and connectivity contrast between t1 and t2 (Supplementary Figure S2). No significant relationship between intraindividual changes in HAM-A/HAM-D and changes in whole-brain rs-FC using bilateral thalamus as the seed were observed.

*Post hoc* analysis adjusting for lifetime exposure to CSHT as a second-level covariate did not significantly affect the rs-FC results, as there was no significant correlation between lifetime exposure to CSHT and rs-FC changes between time points when adjusting for age and HAM-A scores. Moreover, *post hoc* analysis for lateralization effects on rs-FC using a contrast between the right and the left thalamus did not show statistically significant effect.

#### Seed-Based FC Analysis Using a Data-Driven Seed

**FIGURE 2:**
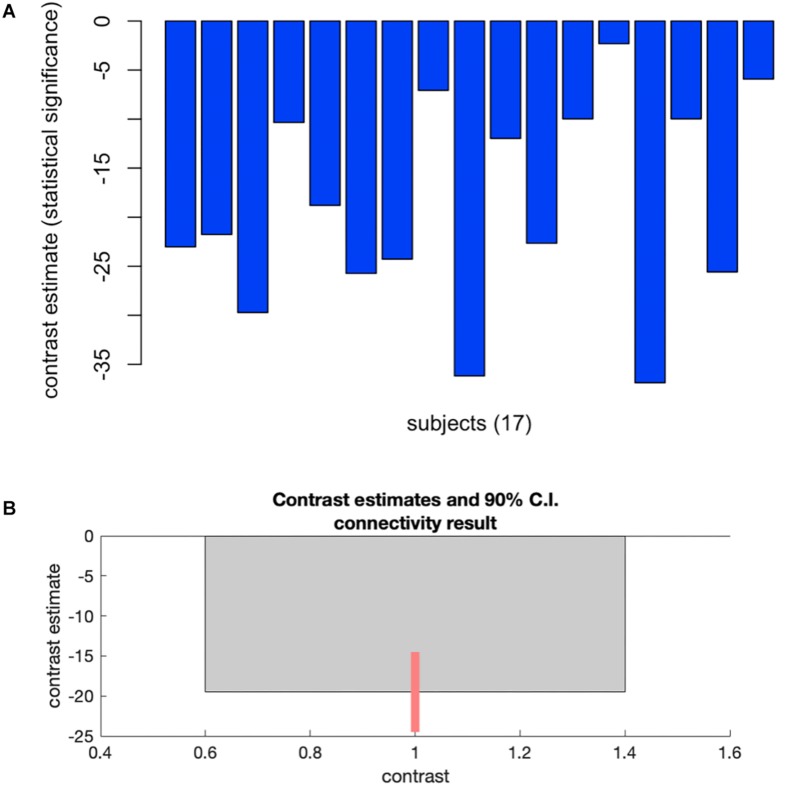
Plots showing changes in whole-brain functional connectivity after a minimum of 60 days of estradiol therapy using multivoxel pattern analysis. **(A)** Plots individual changes in FC between the cluster within the subcallosal cortex and the rest of the brain. **(B)** Shows group average changes in FC (–19.51) between time points, with a 90% confidence interval (C.I: –25.4 to –13.52). Cluster size p-FDR <0.0013.

Group-MVPA identified a cluster within the subcallosal cortex (SubCalC) as the most useful for classification between washout and CSHT (estradiol) conditions; this data-driven seed was used for posterior seed-to-voxel analysis (MNI peak coordinates: −04 + 26 −16; cluster size: 94 voxels; p-FDR: 0.00002). Figure 2 shows individual and group changes in functional connectivity between experimental conditions.

**FIGURE 3:**
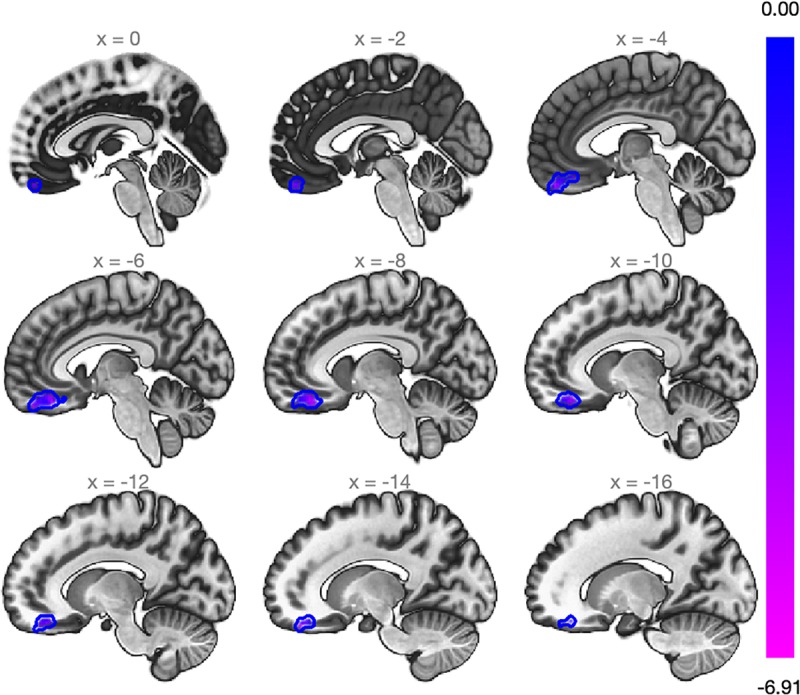
Whole-brain analysis using data-driven seed originated with the MVPA. Slicing view of the cluster within the medial frontal cortex that exhibited a decrease in the rs-FC (beta = –0.27; Cluster size p-FDR = 0.0013). The blue bar on the right decodes statistical significance.

Further whole-brain exploratory analysis using the SubCalC as a seed found a decreased rs-FC (decoupling) between the SubCalC and the medial frontal cortex (MNI peak coordinates: −08 + 38 −20; cluster size: 307 voxels, p-FDR: 0.001) (Figure 3 and Table 3). There was no relationship between intraindividual changes in HAM-A/HAM-D scores and whole-brain connectivity using the SubCalC as a seed.

**TABLE 3:** Seed-to-voxel whole-brain analysis using the subcallosal cortex as a seed.

**Seed**	**Target**	**Cluster size**	**Beta (cluster average)**	**T (cluster average)**	**p-FDR**
SubCalC (−04 + 26 −16)	MedFC (−08 + 38 −20)	307	−0.27	−6.91	0.0013

*The effect of age was controlled. Seed selection was based on previous multivoxel pattern analysis (MPVA). SubCalC, subcallosal cortex; MedFC, medial frontal cortex; FDR, false discovery rate.*

## Discussion

Cross-sex hormone therapy prescribed to transgender women was associated with an increase in the rs-FC between the left thalamus and the left SMC /left putamen after GAS-related gonadectomy. This finding was confirmed by a whole-brain analysis demonstrating an increase in the rs-FC between the left thalamus and a cluster comprising contiguous voxels from the sensory and motor cortices. Further, the data-driven analysis showed a deactivation of a cluster within the subcallosal cortex during estradiol treatment compared to the hypogonadal condition. These findings contribute to the understanding of the relationship between sex hormones and mechanisms of neuroplasticity, since they provide new evidence regarding rs-FC plastic adaptations following the correction of hypogonadal state.

The results also showed a coupling effect of hormone replacement restricted to the left hemisphere (left thalamus – left SMC/left putamen). Although this lateralization effect did not remain statistically significant when left and right thalamus connectivity patterns were directly compared, this finding still deserves attention. Several studies have demonstrated lateral specialization of the basal ganglia nuclei and somatosensory cortex, highlighting the sex differences on lateralization (Pelzer et al., 2016; Reber and Tranel, 2016; Sarasso et al., 2017; Gordji-Nejad et al., 2018). Hence, we cannot rule out the possibility that estradiol therapy influences hemispheric specialization.

To our knowledge, only one previous study by Nota et al. (2017) investigated the effects of CSHT in brain functional connectivity due to hypogonadism, however, they did not study post-gonadectomy individuals. Rather, they induced hypogonadism using a gonadotropin releasing hormone analog (GnRHa) for 4 months. According to their results, resting-state networks were not affected by hormonal status during either GnRHa or GnHRa + CSHT administration. Thus, the study described is not comparable to this present body of work, since the SMC was not included as a region of interest in that study, and the use of GnRHa was considered a potential confound. As a matter of fact, only a few studies have been dedicated to the investigation of sex hormone effects on the sensorimotor circuitry.

### Thalamocorticostriatal Circuit

The thalamus is involved in the integration and modulation of motor and sensory information from (and for) the body (Roelfsema and Holtmaat, 2018). It plays a role as a gateway for contralateral body sensory input, and it is connected to ipsilateral primary sensory and motor cortices (Behrens et al., 2003; Mastropasqua et al., 2014). Immunohistochemical and radiotracer studies have demonstrated that both the thalamus and primary SMC are rich in ER (Taylor and Al-Azzawi, 2000; Osterlund and Hurd, 2001). This evidence provides a reasonable background to support our results; i.e., there is a neuroanatomical and physiological plausibility to explain the increase in the rs-FC observed between these ROIs. In this sense, the coupling between the respective brain areas rich in ER seems to represent a neuroplastic adaptation in the correction of hypogonadism that follows estradiol treatment. The complete absence of circulating sex hormones might result in functional decoupling between these regions, which may contribute to the development of hypogonadal symptoms exhibited post-GAS.

Indeed, Kenna et al. (2008) showed that estrogen therapy in menopause was associated with an augmentation in functional connectivity between the thalamus and the striatum (Kenna et al., 2008). In that study, the authors considered that the increase in functional connectivity was due the protection of cholinergic and dopaminergic neurons after estrogenic therapy. This hypothesis was based on the fact that the two major neurotransmitters involved in passing information through the thalamus are acetylcholine and dopamine, which, respectively, send and receive information to sensory and motor cortex. In accordance with the hypothesis expounded in Kenna et al. (2008), Gardiner et al. (2004) had previously proposed a neuroprotective role for estradiol in the striatal circuit that was related to dopamine transporters. Using single-photon emission computed tomography (SPECT) and [99mTc]TRODAT-1, a radiopharmaceutical that binds to the presynaptic dopamine transporter, they demonstrated that 6 weeks of estrogen therapy increased the number of dopamine transporters in the left putamen (Gardiner et al., 2004).

Moreover, the thalamus and the dorsal striatum have also been implicated in deep learning networks. According to Roelfsema and Holtmaat (2018), part of the deep learning mechanism depends on the activation of the sensory cortex following the reception of sensory input from the thalamic relay. This activation contributes to the establishment of a complex learning process that involves the integration of forward and backward information at different layers of the sensory cortex (Roelfsema and Holtmaat, 2018). In relation to our results, the demonstration of the coupling between the left thalamus and the left putamen/left SMC contributes to the current debate about the potential role of estradiol in synaptic feedback during the learning process. For instance, the better performance in a finger-tapping task following estrogen therapy demonstrated by Bayer and Hausmann (2010) suggests more than only neuroprotective effects over simple motor function (Bayer and Hausmann, 2010; Carpenter et al., 2016). It also suggests additional benefits for motor learning, since learning more complex finger-tapping tasks depends on incorporating information that arrives from the SMC (Herrero et al., 2002). Thus, there seems to be a close relationship between estrogen therapy and functional coupling in the sensorimotor-thalamus-striatum circuit.

### Connectivity in Subcallosal and Medial Frontal Cortices

Finally, using a classificatory analysis with group-MVPA, we identified a decreased activity within the SubCalC after 60 days of estradiol administration. The SubCalC lies under the anterior cingulate cortex and receives input from the limbic lobe. It mediates cognitive and emotional processing – making it a central component of emotional regulation (Mayberg, 2008). It has also been claimed to be a potential biomarker to predict neurocognitive outcomes during treatment of depressive disorders (Dunlop et al., 2017), as well as a potential target for treatment of resistant depression (Li et al., 2017; McInerney et al., 2017). Using the SubCalC as a seed to investigate rs-FC in a seed-to-voxel analysis, we found it to be functionally decoupled from the medial frontal cortex (MedFC) following estradiol administration. Interestingly, deactivation of the SubCalC and portions of the medial frontal cortex was also demonstrated following recovery from depressive symptoms in individuals who responded to cognitive behavior therapy (Goldapple et al., 2004; Li et al., 2017).

During the gender-affirming process, there is an improvement in depressive symptoms and a reduction in suicidal ideation associated with the beginning of CSHT (Gorin-Lazard et al., 2013; da Silva et al., 2016; Tucker et al., 2018). Although these improvements can be partially assumed to be a consequence of the reduction of the body incongruence once CSHT has started, they might also owe to changes in functional connectivity of areas involved in emotions and cognition. Comparatively, in non-transgender individuals, shifts in estradiol and testosterone levels were demonstrated to negatively impact mood (Gordon et al., 2018; Ng et al., 2018), anxiety (Hwang et al., 2015), emotional regulation (Syan et al., 2017; Dan et al., 2018), and cognitive processes (Maki et al., 2001; Berent-Spillson et al., 2010). In this sense, there is growing evidence supporting the existence of a relationship between behavioral, affective and cognitive measures, and changes in brain anatomy and/or functionality (Berent-Spillson et al., 2010; Albert et al., 2015; Syan et al., 2017, 2018; Weis et al., 2017; Dan et al., 2018).

Considering the evidence of increased risk for mood and anxiety disorders in men and women after deprivation of sex hormones, our data point to an association between estradiol administration and rs-FC adaptations involved in emotional and cognitive processes. The emotional improvement seen following the beginning of CSHT might be linked to the deactivation of the SubCalC or to the decoupling between the SubCalC and the MedFC (Gorin-Lazard et al., 2013; Tucker et al., 2018). Further studies are needed to better understand the effects of CSHT on mood and emotional regulation in the transgender population. Nevertheless, the contrast between washout and CSHT examined here provides a unique model, since very low to absent concentrations of sex hormones are found after gonadectomy. This allows us to investigate the impact of CSHT on the brain without the interference of native gonadal hormones.

Importantly, hormone therapy for transgender women is currently limited to the same estradiol formulations worldwide, such as oral estradiol valerate or non-oral transdermal (gel or patches) 17β-estradiol. Some countries also have parenteral estradiol valerate or cypionate for intramuscular administration while others, including Brazil, may still use oral conjugated equine estrogens. Dosages are individualized according to clinical response. Women who had already been submitted to GAS receive estrogen-only treatment (no anti-androgens are added to estrogen therapy).

This study is the first longitudinal study demonstrating the impact of CSHT on the rs-FC of the somatosensory cortex after GAS. In addition, all participants were not taking psychotropic medications at baseline, as well as they did not meet current criteria for major psychiatric disorders. Moreover, ROI-to-ROI and seed-to-voxel whole-brain analyses were convergent in their findings regarding the SMC. However, the strengths highlighted above were accompanied by some limitations which must be considered. For instance, although heart and respiratory rates were not altered while HAM-A was assessed, these parameters were not measured during MRI acquisition. Moreover, the lack of a control group and of appropriate scales to evaluate hypogonadal symptoms must also be taken into consideration. The present study was completed using a within-subjects design (self-controlled) and therefore lacks a comparison group. Ideally, a control group should include transwomen left on washout for more 60 days after 30 days of washout (t1), instead of reintroducing CSHT for new assessments at time point two (t2). Although the absence of a comparison group reduces the specificity of our results, this decision was made due to ethical considerations, as exposing transgendered women to a longer washout period for experimental purposes would result in prolonged exposure to hypogonadism and its consequences. The lack of relationship between the behavioral measures and changes in rs-FC might be partially due to which specific brain areas were priori defined on this study. Further studies regarding hypogonadism should also take into consideration the function of brain areas related to mood and anxiety by employing more specific scales for the phenomena under investigation. The sample size is modest, which also limits our ability to perform secondary analyses. Further, this is a pilot study to test the modulation of CSHT over the somatosensory network in transgender women post-GAS, and these results must be carefully analyzed when extrapolating them to transgender men. Larger studies employing complementary network measure are also needed to fully explore the network effects of estradiol on brain connectivity.

In conclusion, in this study, we found that CSHT in transgender women after GAS impacted rs-FC over the 3 major systems of the brain: cognitive, emotional and sensorimotor. Sensorimotor networks and subcallosal cortex seem to respond to estradiol administration to compensate for deprivation of sex hormones after gonadectomy in transgender women. These results suggest that there is a link between CSHT, mental health, and neuroplasticity in transgender people.

## Data Availability

The datasets generated for this study are available on request to the corresponding author.

## Ethics Statement

This project was approved by the Ethics Review Board of the Hospital de Clínicas de Porto Alegre (CEP 15-0199). All participants gave written informed consent according to the Declaration of Helsinki.

## Author Contributions

MS, ÂC, and ML designed the study. MS, MA, and KS acquired the neuroimage. MS, AF, DS, and CG recruited the participants. MS, AF, DS, CG, FC, and KS acquired the clinical data. PS and TF performed the endocrinological consultations. FC and JC performed the psychological evaluation. MS, SS, LM, and BF performed the neuroimaging analysis. MS, PS, and ÂC carried out the statistical analysis. MS, SS, PS, ML, MA, LM, BF, and JC wrote and critically revised the manuscript. All authors read and approved the final version of the manuscript.

## Conflict of Interest Statement

The authors declare that the research was conducted in the absence of any commercial or financial relationships that could be construed as a potential conflict of interest.

## Footnotes

1 http://www.R-project.org

## Supplementary Material

FIGURE S1 Bar plots **(A,B)** show intraindividual changes in Hamilton’s depression and anxiety rating scores from time point 1 to time point 2 (t2–t1).

FIGURE S2 Blue shows brain regions that exhibit decoupled connectivity with the left thalamus, while red indicates coupled connectivity with the left thalamus. **(A,B)** represent respectively, whole-brain connectivity analysis using left thalamus as seed at t1 and t2, respectively, while **(C)** show significant changes on functional connectivity between t1 and t2. Statistical threshold at peak-height <0.001 and size p-FDR <0.05.
