# Supplementary figures and images for: Effects of Estradiol Therapy on Resting-State Functional Connectivity of Transgender Women After Gender-Affirming Related Gonadectomy
Source: Front Neurosci. 2019 Aug 7;13:817. doi: 10.3389/fnins.2019.00817 (PMC6692765; doi:10.3389/fnins.2019.00817)

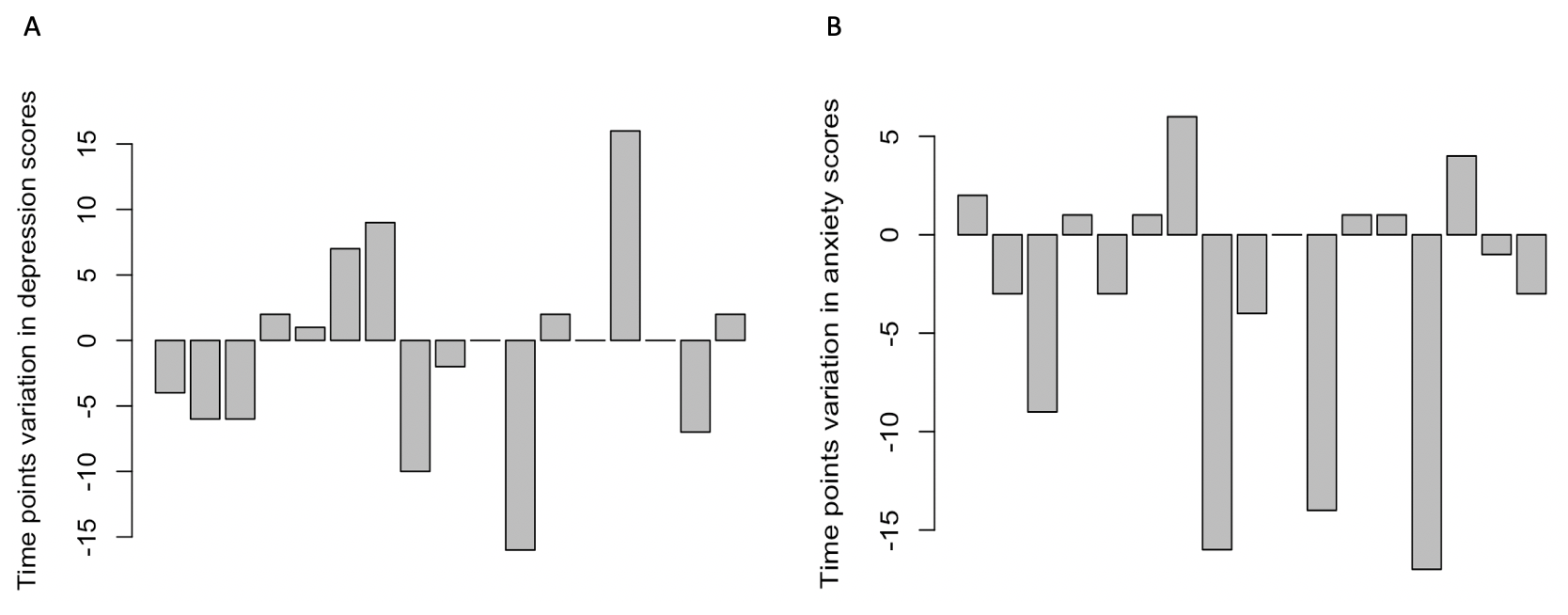

Supplement: FIGURE S1 — Bar plots (A,B) show intraindividual changes in Hamilton’s depression and anxiety rating scores from time point 1 to time point 2 (t2–t1). [file Image_1.PNG]

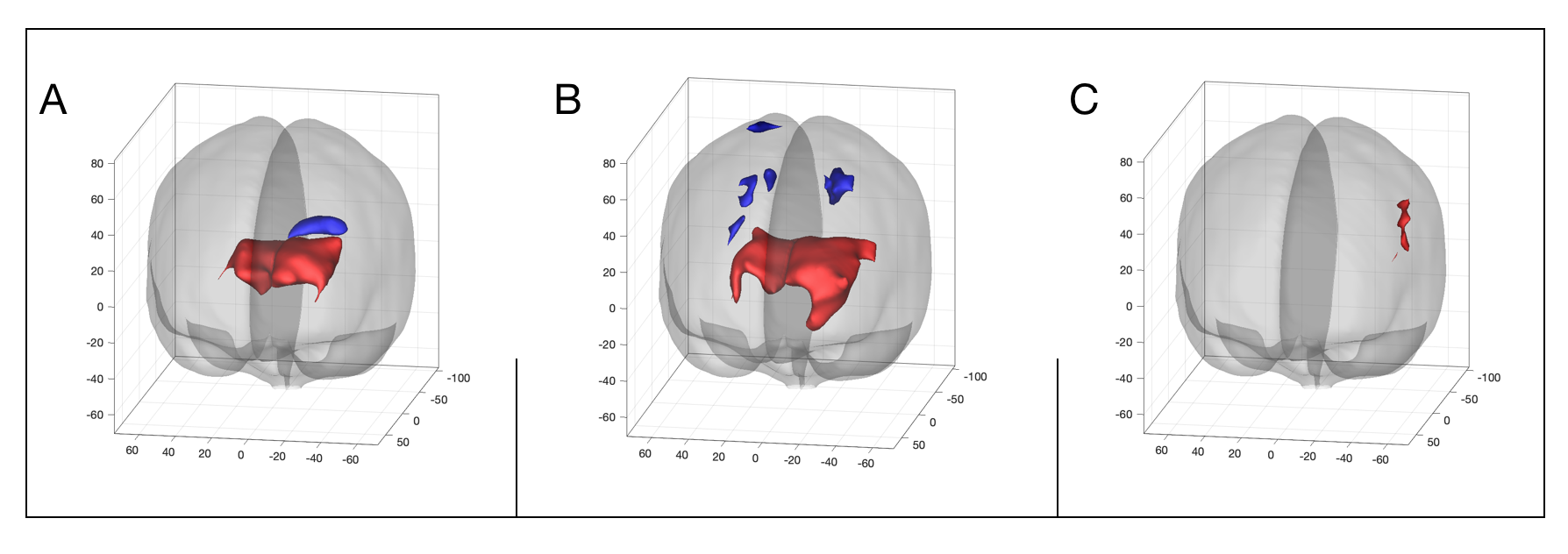

Supplement: FIGURE S2 — Blue shows brain regions that exhibit decoupled connectivity with the left thalamus, while red indicates coupled connectivity with the left thalamus. (A,B) represent respectively, whole-brain connectivity analysis using left thalamus as seed at t1 and t2, respectively, while (C) show significant changes on functional connectivity between t1 and t2. Statistical threshold at peak-height <0.001 and size p-FDR <0.05. [file Image_2.PNG]
